# Prevalence of Risk Factors Associated With Mental Health Symptoms Among the Outpatient Psychiatric Patients and Their Family Members in China During the Coronavirus Disease 2019 Pandemic

**DOI:** 10.3389/fpsyg.2021.622339

**Published:** 2021-05-25

**Authors:** Yan Qiu, Jing Huang, Jinghui Sun, Jiaxu Zhao, Apian Chen, Jindong Chen, Renrong Wu, Sujuan Li, Ziwei Teng, Yuxi Tan, Bolun Wang, Haishan Wu

**Affiliations:** ^1^Department of Psychiatry, National Clinical Research Center for Mental Disorders, China National Technology Institute on Mental Disorders, The Second Xiangya Hospital of Central South University, Changsha, China; ^2^Department of Psychology, School of Educational Science, Hunan Normal University, Changsha, China; ^3^Department of Radiology, The Second Xiangya Hospital of Central South University, Changsha, China

**Keywords:** psychiatric patients, family members, depression, anxiety, acute stress, COVID-19

## Abstract

**Objective:** To investigate the prevalence of and risk factors associated with mental health symptoms in psychiatric outpatients and their family members in China during the COVID-19 pandemic.

**Methods:** This cross-sectional, survey-based, region-stratified study collected demographic data and mental health measurements for depression, anxiety and acute stress from 269 psychiatric patients and 231 family members in the Second Xiangya Hospital in China from April 27, 2020 to May 8, 2020. Binary logistic regression analysis was performed to identify risk factors associated with mental health outcomes.

**Result:** The results of this survey revealed that symptoms of depression, anxiety, and acute stress were highly prevalent symptoms in the psychiatric patient group. Respondents who were female, unmarried or highly educated were significantly more likely to have the above symptoms. In the family member group, more than half of them felt that the burden of nursing had increased during the epidemic. Subjects with a high degree of burden of care were significantly more likely to exhibit the above mental health symptoms, while females were significantly more likely to have acute stress.

**Conclusions:** The results of this survey revealed a high prevalence of mental health disorder symptoms among psychiatric patients and an increased burden of nursing among their family members after the COVID-19 outbreak in China. Understanding the risk factors in those particular groups of people help improve the public health service system for mental health problems during public health events. For further study, exploration of the needs of mental health services and dynamic change tracking will be needed.

## Introduction

Coronavirus Disease 2019 (COVID-19), a highly infectious disease characterized by pneumonia and complications like acute respiratory distress syndrome, broke out in December 2019 (JHU, 2020; Khan et al., 2020a). More than 10 million people have been diagnosed globally, including about 80,000 cases in China (Khan et al., 2020b). Following the timely response, the current status of prevention and control in China has become relatively stable, and even places with a high risk of infection, such as hospitals, have also reopened and restored their functions under strict quarantine rules. The COVID-19 pandemic has increased the risk of mental illness, such as anxiety, depression, and other mental disorders, as well as changed people's daily routines, including sleep, exercise, work, or medical treatment (Khan et al., 2021; Nakamura et al., 2021). During the early stages of the COVID-19 epidemic, global attention was mainly focused on infected patients, frontline medical staff and populations in some special stages. Previous researchers had reported that infected individuals had an increased risk of mental illness, that frontline medical staff had greater occupational hazards and stress than other medical staff, that adolescents had a low perception of their susceptibility to and the seriousness of COVID-19, and that pregnant women displayed a decreased level of physical activity and quality of life (Biviá-Roig et al., 2020; Commodari and La Rosa, 2020; Khan et al., 2020c; Zhang et al., 2020). However, some marginalized groups of people might have been neglected, such as patients with mental disorders and/or other chronic diseases (Wright et al., 2020).

Although 173 million people in China are suffering from mental illnesses, it is still common to see psychiatric patients being neglected and discriminated against (Xiang et al., 2012). When an epidemic occurs, people with mental disorders are usually more susceptible to infection due to a poor awareness of the risk of spreading, the confined conditions in psychiatric wards, as well as diminished efforts regarding personal protection for patients (Kim et al., 2019). It was reported that large-scale COVID-19 nosocomial infections occurred in Wuhan Mental Health Center as well as a psychiatric hospital in South Korea (Ji et al., 2020). In addition to the inpatients hospitalized in mental health institutions, most psychiatric patients are stable and living in the community. Due to national travel and quarantine regulations, those psychiatric patients who should have received regular evaluation and medication in outpatient clinics did not receive such care. Even though emergency service systems such as remote consultation, online consultation, and medication delivery *via* mail have been launched to provide services for community psychiatric patients (Li et al., 2020), it is still far from meeting their demands. Compared with the normal population, these community patients with mental health problems are more sensitive to stress from COVID-19 and more susceptible to emotional reactions related to COVID-19, which may lead to recurrence or deterioration of existing mental health problems (Melamed et al., 2020). So far, the emotional changes these patients have encountered and their respective impacts are rarely reported. Ignoring the impact of the epidemic on people with mental illnesses will not only increase the difficulty of the prevention and control of COVID-19 but also exacerbate the existing issue of health care inequalities.

Patients with long-lasting severe mental disorders are frequently found unable to fulfill typical roles expected by society at their age and intellectual ability (Dziwota et al., 2018). Most people with mental illnesses are now undergoing community care from their family members. Due to the stigma of psychiatric disorders, family members of psychiatric patients are often discriminated against and have an inferiority complex, which makes them unknowingly avoid social activities or change their lifestyles, thus greatly impacting their lives (van der Sanden et al., 2016). In addition, psychiatric illness and distress bring heavy psychological pressure and financial burden to families of psychiatric patients, and heavy care work also increases their physical exertion, which is prone to cause various emotional disorders (Niu and Zhang, 2020).

Due to strict social distancing rules, face-to-face investigations were more often replaced with online data collection in previous studies. However, concerns have been expressed about the selection bias regarding online data collection. One study reported that significantly more young people and mildly ill patients were recruited due to the switch of investigation method from offline to online (Hao et al., 2020). To reduce selection bias, this study issued paper questionnaires on site to investigate and evaluate the emotional changes and psychological shocks in psychiatric patients and their family members. As one-third of the general population in China exhibited symptoms of depression or anxiety due to the impact of COVID-19 (Wang et al., 2020a), we hypothesized that depression or anxiety of greater severity could have happened in patients with mental illness and their family members. This survey aims to reveal the characteristics of mental health needs in psychiatric patients and their family members during the COVID-19 epidemic and also help to improve psychiatric services in case of other future disease epidemics.

## Methods

### Participants

Participants (patients and family members) were recruited at the psychiatric outpatient department of the Second Xiangya Hospital of Central South University, China, from April 27 to May 8, 2020, when the hospital had just begun its full resumption of outpatient service. Four trained researchers conducted recruitment among patients and family members waiting in outpatient clinics using convenience sampling. Written informed consent was obtained from all patients and their family members. The authors assert that all procedures contributing to this work comply with the ethical standards of the relevant national and institutional committees on human experimentation and with the Helsinki Declaration. All procedures involving human subjects/patients were approved by the Ethics Review Committee of Second Xiangya Hospital of Central South University (No. LYF2020125).

### Inclusion and Exclusion Criteria

All psychiatric patients must be aged 16 years or above and be previously diagnosed by psychiatrists to suffer from F20 Schizophrenia, F22 Persistent delusional disorders, F23 Acute and transient psychotic disorders, F30 Manic episodes, F31 Bipolar affective disorders, F32 Depressive episodes, F33 Recurrent depressive disorders, F41 other anxiety disorders (including F41.1 generalized anxiety disorders, F41.0 panic disorders, F41.2 mixed anxiety and depressive disorders), F42 Obsessive-compulsive disorder, or F43 Reactions to severe stress, and adjustment disorders based on the 10th revision of the International Statistical Classification of Diseases and Related Health Problems (ICD-10) criteria. Selected family members of the psychiatric patients were aged 18 years or above and did not suffer from psychiatric illnesses, who had caregiving relationship with patients. Exclusion criteria included inability to complete a survey, presence of severe chronic medical disorders (including neurological, cardiovascular, respiratory, endocrine, and inflammatory disorders) and suspected/confirmed cases of COVID-19.

### Measures

A paper questionnaire was administered to all participants. The structured questionnaire consisted of questions that covered several areas: (1) demographic data; (2) change of medical care for psychiatric patients and burden of care for family members; (3) Impact of Event Scale-Revised items (IES-R); (4) Patient Health Questionnaire-2 items (PHQ-2); (5) Generalized Anxiety Disorder-2 items (GAD-2).

Demographic data was self-reported by the participants, including gender (male or female), age (16–19, 20–35, 36–40, or >40 years), place of residence (urban or rural), marital status (married or unmarried), educational level (senior middle school or below, college or vocational school, bachelor degree or above), occupation (student, employed, or unemployed), change of treatment and way of getting medical care (only for psychiatric patients), and burden-of-care degree (only for family members). Diagnosis information was collected from medical records and self-reports of patients or their family members.

We focused on symptoms of depression, anxiety, and distress for all participants, using Chinese versions of validated measurement tools as follows. The PHQ-2 is a simplified questionnaire based on the Patient Health Questionnaire-9 items. Mainly used for screening depression, the PHQ-2 only rates depression as having two core symptoms (low mood and loss of interest) with a cutoff value of no <3, sensitivity of 0.97, and specificity of 0.67 (Maurer, 2012). The GAD-2, developed from Generalized Anxiety Disorder-7 items, was used to screen anxiety disorders with two core symptoms (anxiety and uncontrollable worry) with a cutoff value of no <3, sensitivity of 0.88, and specificity of 0.61 (Cano-Vindel et al., 2018). The Impact of Event Scale-Revised (IES-R) was adopted to measure symptoms of post-traumatic stress disorder (PTSD) during the COVID-19 pandemic (Li, 2020). The IES-R is a self-administered questionnaire that has been well-validated in the Chinese population for determining the extent of psychological impact after exposure to a public health crisis within 1 week of exposure (Wu and Chan, 2003).

### Statistical Analysis

Data analysis was performed using SPSS statistical software version 25.0 (IBM Corp). The significance level was set at α = 0.05, and all tests were 2-tailed. The original scores of the three measurement tools were not normally distributed, so this data was presented as medians with interquartile ranges (IQRs). The ranked data from the counts of each level for symptoms of depression, anxiety, and distress were presented as numbers and percentages. The R^*^C Chi-square tests were applied to compare the prevalence of depression, anxiety, and acute stress symptoms in two populations by demographic characteristics and epidemic-related factors. To determine potential risk factors for symptoms of depression, anxiety, and distress in participants, binary logistic regression analysis was performed. The associations between risk factors and outcomes were presented as odds ratios (ORs) with 95% confidence intervals adjusting for confounders including sex, age, place of residence, marital status, educational level, occupation, and psychiatric diagnosis (only for patients).

## Results

### Demographic Characteristics

Of the 298 psychiatric patients, 269 (90.3%) with a mean age of 27.06 (±11.90) years completed the survey. One hundred and twenty-two participants (45.4%) were aged 20–34 year, 168 (62.5%) were female, 187 (69.5%) were unmarried, 91 (33. 8%), were living in the city, 116 (43.4%) were students, and 81 (30.1%) had a bachelor degree level of education or above. The majority of respondents had: bipolar disorder (36.4%), schizophrenia (14.9%), major depression disorder (24.2%), or anxiety disorders (12.3%). Of the 252 family members who were approached, 231 (91.7%) with a mean age of 41.61 (±10.42) years completed the survey. One hundred and six (45.9%) were aged 35–49 years, 129 (55.8%) were female, 191 (82.7%) were married, 83 (35.9%), were living in the city 57 (24.7%) had a bachelor degree level of education or above, and 133 (57.6%) were unemployed (Table 1).

**Table 1:** Descriptive statistics of demographic characteristics and epidemic-related information for the psychiatric patients and their family members.

	**Patients (*N* = 269) *n* (%)**	**Family members (*N* = 231)*n* (%)**	***P*-value**
**Age**			
16–19	92 (34.2)	4 (1.7)	<0.001
20–34	122 (45.4)	56 (24.2)	
35–49	33 (12.3)	106 (45.9)	
50–65	22 (8.2)	56 (24.2)	
66–68	**/**	9 (4.0)	
**Gender**			
Male	97 (36.1)	98 (42.4)	0.138
Female	168 (62.5)	129 (55.8)	
Unfilled	4 (1.5)	4 (1.7)	
**Marital status**			
Unmarried	187 (69.5)	32 (13.9)	<0.001
Married	79 (29.4)	191 (82.7)	
Unfilled	3 (1.1)	8 (3.5)	
**Urban and rural sources**			
City	91 (33.8)	83 (35.9)	0.884
Town	83 (30.9)	55 (23.8)	
Countryside	83 (30.9)	79 (34.2)	
Unfilled	12 (4.5)	14 (6.0)	
**Education level**			
Senior middle school or below	134 (49.8)	126 (54.5)	0.387
College or vocational school	47 (17.5)	42 (18.2)	
Bachelor degree or above	81 (30.1)	57 (24.7)	
Unfilled	7 (2.6)	6 (2.6)	
**Occupation**			
Student	116 (43.1)	9 (3.9)	<0.001
Employed	77 (28.6)	74 (32.0)	
Unemployed	63 (23.4)	133 (57.6)	
Unfilled	13 (4.8)	15 (6.0)	
**Psychiatric diagnosis**			
Bipolar disorder	98 (36.4)	58 (25.1)	N/A
Schizophrenia	40 (14.9)	49 (21.2)	
Major depression disorder	65 (24.2)	57 (24.7)	
Anxiety disorder	33 (12.3)	15 (6.5)	
Other psychiatric diagnosis	26 (9.7)	40 (17.3)	
Unknown	6 (2.2)	12 (5.0)	
**Relationship**			
Parent	96 (41.6)	N/A	N/A
Spouse	37 (16.0)		
Child	49 (21.2)		
Daughter-in-law or son-in-law	4 (1.7)		
Brother or sister	25 (10.8)		
Other	15 (6.5)		
Unfilled	5 (2.2)		

Approximately 60% of the patients had no change in medicine during the epidemic, while nearly 30% reduced their doses or stopped taking medicine without consulting their psychiatrists. Up to 40% of the patients failed to see their psychiatrists, in person but 13.8% of them obtained medical advice from doctors online. More than half of the patients' family members reported an increase in the burden of care (Table 2).

**Table 2:** Factor details of epidemic-related influence on change of treatment, way of getting medical care for psychiatric patients and burden of care change for their family members.

**Characteristics**	***n* (%)**
**For patients**	
Medical treatment	
Take the medicine regularly without changing the dose	150 (55.8)
Take the medicine regularly and reduce it by yourself	24 (8.9)
Take the medicine regularly and reduce it following doctor's advice	15 (5.58)
Stop taking the medicine by yourself	49 (18.2)
Others	29 (10.8)
Unfilled	2 (0.7)
Method of getting medical care	
Psychiatric specialist hospital	60 (22.3)
General hospital	37 (13.8)
Internet hospital	37 (13.8)
Without follow-up by doctor	110 (40.9)
Others	17 (6.3)
Unfilled	8 (2.9)
**For family members**	
Burden of caring patients	
No increase	65 (28.1)
Mildly increase	79 (34.2)
Moderately increase	41 (17.7)
Severely increase	18 (7.8)
Extremely Severely increase	10 (4.3)
Unfilled	18 (7.8)

### Prevalence of Depression, Anxiety, and Acute Stress

Thirty percentage of psychiatric patients suffered depression, 27.8% anxiety, and 27.8% acute stress. The median scores for depression, anxiety, and acute stress were 2, 2, and 22, respectively (Table 3).

**Table 3:** Factor scores of PHQ-2, GAD-2 and IES-R and prevalence of depression, anxiety, and acute stress symptoms between patients and family members.

**Characteristics**	**Patients**		**Family members**	
	**Median (IQR)**	***N* (%)**	**Median (IQR)**	***N* (%)**
**Total PHQ-2 score**	2 (0.3)	80 (30.0)	0 (0.1)	20 (8.6)
Item 1: Feeling down/depressed/hopeless	1 (0.2)		0 (0.1)	
Item 2: Little interest in doing things	1 (0.2)		1 (0.2)	
**Total GAD-2 score**	2 (0.3)	74 (27.8)	0 (0.2)	25 (10.8)
Item 1: Feeling nervous/anxious/on edge	1 (0.2)		0 (0.1)	
Item 2: Not being able to stop worrying	1 (0.2)		0 (0.1)	
**Total IES-R score**	22 (9, 36.5)	74 (27.8)	11 (3.24)	25 (10.8)
Part 1: Intrusive reaction	7 (2.12)		5 (1.9)	
Part 2: High vigilance	7 (2.12)		3 (0.6)	
Part 3: Avoidance response	7 (2.13)		4 (1.10)	

Univariate analyses showed that depression symptoms were more severe among participants who were female, <20 years, unmarried, and primary or lower secondary school students [e.g., depression among female vs. male: 62 [36.9%] vs. 17 [17.5%]; *P* = 0.001]. Females and primary or lower secondary school students also reported experiencing higher levels of anxiety [e.g., anxiety among female vs. male: 54 [32.1%] vs. 19 [19.6%]; *P* = 0.028]. Distress levels were found to be higher among females, primary or lower secondary school students, and patients with bipolar disorder [e.g., acute stress among female vs. male: 59 [35.1%] vs. 15 [15.5%]; *P* = 0.001] (Supplementary Tables 1, 2).

8.6% of family members suffered from depression, 10.8% suffered from anxiety, and 10.8% suffered from acute stress. No significant subgroup differences were observed in family members. The median scores for depression, anxiety, and acute stress was 0, 0, and 11, respectively (Table 3).

### Factors Associated With Depression, Anxiety, and Acute Stress

Binary logistic regression analyses showed that gender (female) and education level were risk factors for depression, anxiety, and acute stress among psychiatric patients. Burden-of-care degree was an independent factor among family members for the three conditions above, while gender (female) was also an independent factor for distress. The detailed results of the logistic analyses are shown in Table 4.

**Table 4:** Binary logistic regression analysis^Δ^ of risk factors associated with symptoms of depression, anxiety, and acute stress.

**Variables**	**OR (95% CI)**	***P*-value**
*Models for depression*		
Patients		
Gender (female vs. male)	3.640 (1.706, 6.765)	0.001
Marital status (unmarried vs. married)	2.490 (1.164, 5.324)	0.019
Education level (Group 1 vs. Group 2)^*^	4.105 (1.335, 12.624)	0.014
Education level (Group 3 vs. Group 2)^*^	4.168 (1.302, 13.347)	0.016
Family members		
Burden of care (score between 0 and 4)	2.187 (1.455, 3.289)	<0.001
*Models for anxiety*		
Patients		
Gender (female vs. male)	3.173 (1.490, 6.756)	0.003
Education level (Group 1 vs. Group 2)^*^	4.897 (1.402, 17.106)	0.013
Education level (Group 3 vs. Group 2)^*^	6.507 (1.806, 23.447)	0.004
Family members		
Burden of care (score between 0 and 4)	2.186 (1.486, 3.216)	<0.001
*Models for acute stress*		
Patients		
Gender (female vs. male)	3.271 (1.556, 6.875)	0.002
Education level (Group 1 vs. Group 2)^*^	1.738 (0.668, 4.520)	0.257
Education level (Group 3 vs. Group 2)^*^	3.634 (1.369, 9.646)	0.010
Family members		
Gender (female vs. male)	3.817 (1.272, 11.455)	0.017
Burden of care (score between 0 and 4)	2.341 (1.545, 3.546)	<0.001

* *Education level: Group 1: Senior middle school or below, Group 2: College or vocational school, Group 3: Bachelor degree or above*.

Δ *The regression models for patients included as independent variables: age, gender, marital status, education level, occupation, psychiatric diagnosis, medical treatment and method of getting medical care. The backward selection method was then applied to remove all insignificant variables*.

*The regressions for family members included as independent variables: age, gender, marital status, education level, occupation, relationship and burden of care degree, with the backward selection method then applied to remove all insignificant variables*.

In depression models, female (OR, 3.640, 95% CI, 1.706–6.765; *p* = 0.001), unmarried (OR, 2.490; 95% CI, 1.164–5.324; *p* = 0.019), senior middle school or below (OR, 4.105; 95% CI, 1.335–12.624, *p* = 0.014) and bachelor degree or above (OR, 4.168; 95% CI, 1.302–13.347, *p* = 0.016) were selected as independent factors among psychiatric patients.

Two variables were independently associated with anxiety risk factors: gender (female) (OR, 3.173; 95% CI, 1.490–6.756; *p* = 0.003) and education level including senior middle school or below (OR, 4.897, 95% CI, 1.402–17.106; *p* = 0.013), and bachelor degree or above (OR, 6.507; 95% CI, 1.806–23.447; *p* = 0.004).

For acute stress symptoms, psychiatric patients had two risk factors: gender (female) (OR, 3.271; 95% CI, 1.556–6.875; *p* = 0.002) and education level for only bachelor degree or above (OR, 3.634; 95% CI, 1.369–9.646; *p* = 0.010).

## Discussion

To the best of our knowledge, this is the first survey on the mental health of psychiatric patients and their family members during the COVID-19 epidemic in China. Our findings present concerns about the psychological well-being of psychiatric patients and their family members during the outbreak of COVID-19. The current study indicates that many psychiatric patients and family members might have experienced several mental health problems and changes in medical treatment or care burden during the epidemic. These findings can provide indirect evidence to other areas of China and other countries to help reduce depression, anxiety and acute stress in psychiatric patients and their family members.

This cross-sectional survey revealed a high prevalence of mental health symptoms among psychiatric patients after the COVID-19 outbreak in China. The prevalence of emotional symptoms and stress symptoms in patients with mental illness found in this study is equivalent to that of another general population investigated in a preliminary online survey in late January 2020, where nearly one-third of the respondents had experienced moderate to severe mental health conditions (Wang et al., 2020a). Another epidemiological survey conducted among the general population in China in early February 2020 also shows that nearly 35% of the respondents displayed psychological distress during the COVID-19 epidemic (Yuan et al., 2020). Although the investigation time of this study was after the peak of the epidemic in China, the incidence of emotional problems among mentally ill patients remains the same. Following this logic, we speculate that psychiatric patients might have experienced more severe symptoms of anxiety, depression, and stress during the peak of the epidemic compared to the healthy population. Several possible facts can support this speculation. Firstly, that strict social isolation and decreased social activities affect neuroendocrine function (Wang et al., 2020c), possibly increasing the risk of suicide and stress-related aggression in this group of people (Calati et al., 2019; Brooks et al., 2020). Secondly, that social isolation also prevents patients from receiving necessary medical care, aggravating their original psychiatric conditions (Vieta et al., 2012). Thirdly, that despite the introduction of virtual medical care through the internet, its effectiveness is not as much as medical care in person. During the COVID-19 epidemic, a lack of health care in person and failure to administer timely treatment may have caused fluctuations in patients conditions (Li et al., 2020).

Binary logistic regression analysis confirmed that after controlling confounding factors, gender and education level were independent risk factors for depression, anxiety and stress. Among patients with mental illness, women were more susceptible to depression, anxiety and acute stress. It has been shown that with different levels of response to stress, women are more sensitive to the release of corticotropin-releasing factor (CRF) (Bangasser and Wiersielis, 2018). Inappropriate or persistent CRF release is strongly linked to depression and anxiety (Vasconcelos et al., 2020). In addition, hormonal changes in women during the menstrual cycle or menopause can also lead to more pronounced mood changes (Christiansen and Berke, 2020). Psychosocial stress may affect the hypothalamus-pituitary gland-gonads, affecting the hormone levels and further changing mental states (Nabi et al., 2020). In this study, we found that groups with lower or higher education levels had a higher risk of depression, anxiety and stress for COVID-19. For the group with less education, a poor knowledge reserve and ability to analyze information from social media might have limited their capability of coping with the stress from the epidemic, resulting in psychological stress reactions and even some mental illnesses from excessive stress. However, people who received university education and above could also become nervous and anxious during the epidemic due to excessive attention to information related to the epidemic (Myrick and Willoughby, 2019). People with high-level education tend to have a higher expectation for their jobs and sense of value for society than those with low or medium-level education. The impact of COVID-19 on their working status might have created a psychological gap between expectation and reality, followed by anxiety and depression (Lu et al., 2019). In this regard, online or smartphone-based psychological interventions (such as cognitive behavioral therapy) could be provided to this specific group of people in the hope of reducing the risk of depression and anxiety (El Morr et al., 2020).

The prevalence of emotional symptoms and stress symptoms in family members in this study is much lower than that of people with mental illness in the same period. This shows that people without mental disorders have better emotional self-regulation and resistance to stress than patients. At the same time, we have noticed that this proportion is lower than that investigated during the early epidemic for the general population (Wang et al., 2020b). This may be due to relief brought on by an effective prevention system against COVID-19, easing the psychological impact on people without mental disorders.

Several limitations of the current study should be considered. Firstly, that this is a cross-sectional survey that failed to monitor the changes in the mental health of psychiatric patients during the epidemic. Since the same survey was not conducted on the general public in the same period, it is not enough to compare the prevalence with them. Secondly, that all participants in the current study were recruited from outpatient department in one hospital. Selection bias may have been introduced to this study. Given the diverse geographical environment and the management strategies in different hospitals, the extrapolation of these results to psychiatric patients in other regions remains to be verified. Thirdly, that the data of this study was not taken from the peak period of COVID-19 in China, but 2 months after the peak period. Therefore, it may not accurately reflect the emotional changes of psychiatric patients during the initial outbreak. In addition to the above, in future studies, the impact of the epidemic on these two groups in lifestyle habits needs to be considered comprehensively, as well as mental health symptoms, way of seeking medical care, and change of care burden.

## Conclusion

The results of this study suggest that psychiatric patients and their family members are at risk of depression, anxiety and acute stress symptoms even during the COVID-19 remission period. Given that the global epidemic of COVID-19 is still continuing, it is necessary to follow up these subjects with high-risk factors. From the perspective of psychosocial services, the public health service system for special populations during public health emergencies needs to be further improved. Future research needs to track the characteristics of dynamic changes in mental health and understand the needs of mental health services in this particular group of people.

## Data Availability Statement

The original contributions presented in the study are included in the article/Supplementary Materials, further inquiries can be directed to the corresponding authors.

## Ethics Statement

The studies involving human participants were reviewed and approved by Ethics Review Committee of Second Xiangya Hospital of Central South University. Written informed consent was obtained from each participant after the study was explained.

## Author Contributions

HW, BW, JC, and RW: conception and design. YQ, JS, JZ, and AC: recruitment of subjects. YQ, SL, ZT, and YT: acquisition of data. YQ and JH: analysis and interpretation of data. YQ, HW, and BW: drafting the manuscript. All authors contributed to and have approved the final manuscript.

## Conflict of Interest

The authors declare that the research was conducted in the absence of any commercial or financial relationships that could be construed as a potential conflict of interest.
