# Supplementary material for: Prevalence of Risk Factors Associated With Mental Health Symptoms Among the Outpatient Psychiatric Patients and Their Family Members in China During the Coronavirus Disease 2019 Pandemic
Source: Front Psychol. 2021 May 25;12:622339. doi: 10.3389/fpsyg.2021.622339 (PMC8185154; doi:10.3389/fpsyg.2021.622339)
Supplement: Supplementary file 1 [file Table_1.DOCX]

**SUPPLMENT TABLES**

Table 1 Prevalence of Symptoms of Depression, Anxiety, and Acute Stress in the psychiatric patients by Demographic Characteristics and Epidemic-Related Factors

|  | **Patients(N=269)** | | | | | |
| --- | --- | --- | --- | --- | --- | --- |
|  | PHQ-2  n(%) | *P* | GAD-2  n(%) | *P* | IES-R  n(%) | *P* |
| **Overall** | 80(30.0) | N/A | 74(27.8) | N/A | 74(27.8) | N/A |
| **Gender** |  |  |  |  |  |  |
| Male | 17(17.5) | 0.001* | 19(19.6) | 0.028* | 15(15.5) | 0.001* |
| Female | 62(36.9) |  | 54(32.1) |  | 59(35.1) |  |
| **Age** |  |  |  |  |  |  |
| 16-19 | 39(42.4) | 0.015* | 30(32.6) | 0.578 | 26(28.3) | 0.815 |
| 20-34 | 29(24.0) |  | 29(24.0) |  | 36(29.8) |  |
| 35-49 | 8(25.8) |  | 9(29.0) |  | 7(22.6) |  |
| 50-65 | 4(18.2) |  | 6(27.3) |  | 5(22.7) |  |
| **Marital status** |  |  |  |  |  |  |
| Unmarried | 65(34.8) | 0.005* | 55(29.4) | 0.268 | 55(29.4) | 0.268 |
| Married | 14(17.7) |  | 18(22.8) |  | 18(22.8) |  |
| **Urban and rural sources** |  | | | | | |
| City | 28(30.8) | 0.566 | 29(31.9) | 0.308 | 28(30.8) | 0.812 |
| Town | 27(32.5) |  | 24(28.9) |  | 22(26.5) |  |
| Countryside | 21(25.3) |  | 18(21.7) |  | 23(27.7) |  |
| **Education level** |  | | | | | |
| Senior middle school or below | 44(32.8) | 0.003* | 38(28.4) | 0.001 | 33(24.6) | 0.003* |
| College or vocational school | 4(8.5) |  | 3(6.4) |  | 7(14.9) |  |
| Bachelor degree or above | 28(34.6) |  | 29(35.8) |  | 33(40.7) |  |
| **Occupation** |  | | | | | |
| Student | 46(39.7) | 0.003* | 40(34.5) | 0.014 | 36(31.0) | 0.64 |
| Employed | 18(23.4) |  | 20(26.0) |  | 20(26.0) |  |
| Unemployed | 11(17.5) |  | 9(14.3) |  | 16(25.4) |  |
| **Psychiatric Diagnosis** |  | | | | | |
| Bipolar disorder | 28(28.6) | 0.176 | 28(28.6) | 0.221 | 31(31.6) | 0.021* |
| Schizophrenia | 8(20.0) |  | 6(15.0) |  | 3(7.5) |  |
| Major depression disorder | 27(41.5) |  | 20(30.8) |  | 20(30.8) |  |
| Anxiety Disorder | 9(27.3) |  | 13(39.4) |  | 12(36.4) |  |
| Other Psychiatric Diagnosis | 7(26.9) |  | 7(26.9) |  | 5(19.2) |  |
| **Medical treatment** |  |  |  |  |  |  |
| medicine regularly without changing the dose | 39(26.0) | 0.353 | 36(24.0) | 0.464 | 38(25.3) | 0.256 |
| Take the medicine regularly and reduce it by yourself | 9(37.5) |  | 8(33.3) |  | 6(25.0) |  |
| Take your medicine regularly and follow your doctor's advice | 7(46.7) |  | 6(40.0) |  | 8(53.3) |  |
| Stop taking the medicine yourself | 16(32.7) |  | 16(32.7) |  | 13(26.5) |  |
| others | 10(34.5) |  | 9(31.0) |  | 9(31.0) |  |
| **Method of getting medical care** |  |  |  |  |  |  |
| Psychiatric specialist hospital | 19(31.7) |  | 16(26.7) | 0.667 | 17(28.3) | 0.699 |
| general Hospital | 13(35.1) |  | 13(35.1) |  | 10(27.0) |  |
| Internet Hospital | 11(29.7) |  | 8(21.6) |  | 8(21.6) |  |
| Without follow-up by doctor | 31(28.2) |  | 31(28.2) |  | 35(31.8) |  |
| others | 4(23.5) |  | 3(17.6) |  | 3(17.6) |  |

*p＜0.05

Table 2 Prevalence of Symptoms of Depression, Anxiety, and Acute Stress in the family members by Epidemic-Related Factors

|  | **Family members(N=231)** | | | | | |
| --- | --- | --- | --- | --- | --- | --- |
|  | PHQ-2(%) | *p* | GAD-2(%) | *p* | IES-R(%) | *p* |
| **Overall** | 20(8.6) | N/A | 25(10.8) | N/A | 25(10.8) | N/A |
| **Gender** |  |  |  |  |  |  |
| Male | 9(9.2) | 0.863 | 10(10.20) | 0.734 | 7(7.1) | 0.104 |
| Female | 11(8.5) |  | 15(11.6) |  | 18(13.9) |  |
| **Age** | | | | | | |
| 16-19 | 0(0.0) | 0.831 | 0(0.0) | 0.636 | 0(0.0) | 0.752 |
| 20-34 | 6(10.7) |  | 8(14.3) |  | 7(12.5) |  |
| 35-49 | 8(7.5) |  | 9(8.5) |  | 13(12.3) |  |
| 50-68 | 5(7.7) |  | 6(9.2) |  | 4(6.2) |  |
| **Marital status** | | | | | | |
| Unmarried | 3(9.4) | 0.741 | 5(15.6) | 0.355 | 5(15.6) | 0.355 |
| Married | 16(8.4) |  | 19(9.9) |  | 19(9.9) |  |
| **Urban and rural sources** |  |  |  |  |  |  |
| City | 7(8.4) | 0.868 | 6(7.2) | 0.236 | 9(10.8) | 0.606 |
| Town | 4(7.3) |  | 5(9.1) |  | 4(7.3) |  |
| Countryside | 8(10.1) |  | 12(15.2) |  | 10(12.7) |  |
| **Education level** |  |  |  |  |  |  |
| Senior middle school or below | 8(6.3) | 0.222 | 13(10.3) | 0.559 | 11(8.7) | 0.373 |
| College or vocational school | 3(7.1) |  | 3(7.1) |  | 4(9.5) |  |
| Bachelor degree or above | 8(14.0) |  | 8(14.0) |  | 9(15.8) |  |
| **Occupation** |  |  |  |  |  |  |
| Student | 2(22.2) | 0.178 | 3(33.3) | 0.074 | 1(11.1) | 0.927 |
| Employed | 4(5.4) |  | 8(10.8) |  | 7(9.5) |  |
| Unemployed | 13(9.8) |  | 11(8.3) |  | 15(11.3) |  |
| **Psychiatric Diagnosis** |  |  |  |  |  |  |
| Bipolar disorder | 5(8.6) | 0.449 | 5(8.6) | 0.935 | 6(10.3) | 0.311 |
| Schizophrenia | 6(12.2) |  | 6(12.2) |  | 4(8.2) |  |
| Major depression disorder | 5(8.8) |  | 6(10.5) |  | 5(8.80 |  |
| Anxiety Disorder | 2(13.3) |  | 2(13.3) |  | 5(26.7) |  |
| Other Psychiatric Diagnosis | 1(2.5) |  | 5(12.5) |  | 6(15.0) |  |
| **Relationship** |  |  |  |  |  |  |
| parent | 9(9.4) | 0.919 | 11(11.5) | 0.846 | 12(12.5) | 0.854 |
| spouse | 2(5.4) |  | 3(8.1) |  | 4(10.8) |  |
| child | 5(10.2) |  | 6(12.2) |  | 7(14.3) |  |
| Daughter-in-law or son-in-law | 0(0.0) |  | 0(0.0) |  | 0(0.0) |  |
| brother or sister | 2(8.0) |  | 2(8.0) |  | 1(4.0) |  |
| Other | 2(13.3) |  | 3(20.0) |  | 1(6.7) |  |
| **Burden of care** |  |  |  |  |  |  |
| No change or decrease | 2(3.1) | 0.000 | 3(4.6) | 0.000 | 2(3.1) | 0.001 |
| A little increase | 2(2.5) | * | 4(5.1) | * | 5(6.3) | * |
| Moderate increase | 7(17.1) |  | 5(12.2) |  | 8(19.5) |  |
| Great increase | 6(33.3) |  | 6(33.3) |  | 5(27.8) |  |
| Severe increase | 2(20.0) |  | 4(40.0) |  | 3(30.0) |  |

*p＜0.05
